# Guillain-Barré syndrome associated with myasthenia gravis: Three cases report and a literature review

**DOI:** 10.1097/MD.0000000000018104

**Published:** 2019-11-22

**Authors:** Yayun Cao, Mengcui Gui, Suqiong Ji, Bitao Bu

**Affiliations:** Department of Neurology, Tongji Hospital, Huazhong University of Science and Technology, Wuhan, Hubei Province, China.

**Keywords:** anti-ganglioside antibodies, Guillain-Barré syndrome, myasthenia gravis

## Abstract

**Rationale:**

Myasthenia gravis (MG) and Guillain-Barré syndrome (GBS) are 2 common neurologic autoimmune diseases. Although both the diseases can present with acute or subacute onset of muscular weakness involving the limbs and bulb, the coexistence in the same patient is unusual and rarely described in the literature.

**Patient concerns:**

Three cases of combined MG and GBS at the department of Neurology were described. All the 3 patients developed GBS, who had had MG for 30 years, 6 years, and 6 months, respectively.

**Diagnoses:**

The newly developed GBS was clinically confirmed by the clinical features, electromyographic (EMG) studies, typical albumino-cytologic dissociation in cerebrospinal fluid (CSF), and positive anti-ganglioside antibodies in serum.

**Interventions:**

The 3 patients had been treated with intravenous immunoglobulin (IVIG), or plasma-exchange (PE), or IVIG combined with PE in the acute stage of severe muscle weakness. In light of the MG symptoms, they have received glucocorticoids, oral pyridostigmine, and immunosuppressive agents.

**Outcomes:**

The patient 1 was able to walk longer than 5 m with assistance (Hughes 3). The patient 2 had significantly improved, and completely recovered at the 1-year follow-up (Hughes 0). But unfortunately, the patient 3 was severely disabled and chair-bound at the last interview (Hughes 4).

**Lessons:**

The combination of MG and GBS is quite rare. Limbs and oculo-bulbar weakness are the cardinal manifestations of both the diseases. Although their characteristics are quite different, there are still some difficulties in diagnosing them when they occur in the same patient. Early diagnosis and proper treatment will yield satisfactory prognosis. Further researches are needed to elucidate the pathogenesis of the coexistence.

## 1 Introduction

Myasthenia gravis (MG) is a common neurologic autoimmune disease, affecting the neuromuscular transmission mediated by antibodies-dependent autoimmunity. The disease is clinically manifested as pathologic fatigability and weakness of affected muscles. In severe cases, dysphagia and/or dyspnea may occur. The Guillain-Barré syndrome (GBS), the acute inflammatory demyelinating polyneuropathy, cardinally presents with acute-onset symmetric flaccid limb weakness with changes in sensation to some degree. In severe GBS cases, dysphagia and dyspnea may develop. Anti-ganglioside antibodies may be elevated in some GBS cases, which may be considered as biomarkers. Although both the diseases are the common neurologic disorders caused by dysimmunity, the coexistence was unusual.^[1]^ When the 2 conditions happened, the recognition may be difficult. Here we reported 3 cases of MG who developed GBS, trying to remind the clinicians of recognizing the developed GBS in MG patients.

## 2 Case report

Patient 1: A 52-year-old male was hospitalized because he had experienced fluctuating limb weakness and dysphagia for 30 years and exacerbated for 3 days on January 13, 2015. The diagnosis of MG was established based on the characteristic muscular weakness and fatigability, decremental amplitudes on low-frequent repetitive nerve stimulation (RNS) examination, and a positive response to the intramuscular injection of a bolus of neostigmine sulphate. No thymic abnormalities were detected on the chest computed tomography (CT). After the treatment of oral pyridostigmine and corticosteroids, the symptoms were almost relieved with a minimized manifestation status. The drugs were gradually tapered and discontinued 4 years ago. He had achieved the clinical stable remission with normal acetylcholine receptor (AChR) antibodies for 4 years since than. Three days before admission, the patient had flu-like symptoms and the limb weakness appeared again.

After hospitalization, anti-infectious treatment, oral pyridostigmine and small dosage of prednisone were started. However, the muscle weakness rapidly deteriorated, then evolved into symmetric acute flaccid weakness on the 4 limbs and dyspnea. The myasthenic crisis was initially considered and the intratracheal intubation and mechanical ventilation were performed. After the treatment with intravenous immunoglobulin (IVIG), he improved gradually. A month after admission, the limb strength returned to grade 3/5 without any identifiable triggers, but soon the limbs became weaker again and dropped to grade 0/5 in combination with left peripheral facial paralysis. Electromyographic (EMG) studies showed decreased motor amplitudes with marked reduction velocity on the bilateral peroneal nerves. The decreased rates of F waves induced in the bilateral median, ulnar and tibial nerves (25–50%) were evident. In addition, the sensory potentials were not recorded on the bilateral tibial and peroneal nerves. The cerebrospinal fluid (CSF) exanimation disclosed an elevated albumin content (85 mg/dL) with normal cell counts (albumino-cytolotic dissociation). Furthermore, anti-GM1 antibodies were significantly elevated in the serum. Based on the findings, acute peripheral polyneuropathy especially the GBS was highly suspected. The dyspnea and limb paralysis started to improve quickly after plasma-exchange (PE), in cohort with oral pyridostigmine, prednisone and cyclosporine (Hughes 3).

Patient 2: A 61-year-old male was hospitalized because he had suffered alternative unilateral ptosis and fluctuating limb weakness for 6 years and worsening dysphagia for 2 months on July 14, 2016. He was diagnosed with MG according to the typical clinical presentations, decremental decline of amplitude on RNS and the positive response to an intramuscular injection of a bolus neostigmine sulphate 6 years ago, and no thymic abnormalities was detected. Before that, he had had hyperthyroidosis for many years. After irregular medication of oral pyridostigmine and corticosteroids, he still had minimal myasthenic symptoms. Two months before admission, he was aggravated again with worsening dysphagia and dyspnea.

After admission, he was mechanically ventilated on the prompt. On neurologic examination, the muscle strength of the lower limbs was graded 2/5, with areflexia. The possibility of GBS was suspected. The CSF study yielded no abnormalities and EMG studies disclosed a significantly decreased rate of F waves. The motor amplitudes and motor conduction velocities were in normal ranges with prolonged latencies. The elevated titers of anti-GM1 antibodies and AChR antibodies were detected (>8.0 nmol/L). Finally, the diagnosis of combined MG and GBS was confirmed. After the treatment with IVIG, his symptoms were significantly improved. A year after the discharge, he remained in a stable condition (Hughes 0) on oral pyridostigmine, prednisone and tacrolimus.

Patient 3: A 44-year-old male was transferred from the Department of Thoracic Surgery on July 1, 2016 at our hospital. He had been in a good condition until 6 months ago, when ocular type of MG was diagnosed. The chest CT revealed a large anterior mediastinal mass. A biopsied examination suggested the malignant thymoma. Subsequently, chemotherapy (ADOC regimen consisted of cyclophosphamide, doxorubicin, cisplatin, and vincristine) was performed at the Department of Oncology. Two months ago, the patient underwent complete resection of the thymoma (type C, World Health Organization Classification of thymoma). After the surgery the patient remained very well. A month after the operation, he developed a confusional state and dyspnea during the hospitalization. After mechanical ventilation, he had regained his consciousness. But he still had severe limb weakness and dyspnea needing mechanical ventilator even after the introduction of IVIG, thus he was referred to the Neurology Department.

The neurologic test showed the bilateral ptosis, muscle strength graded 3/5 with low muscular tension and absent tendon reflex without alterations in sensations. The patient had received standardized treatments for MG crisis for 1 month, but his symptom had not improved significantly. EMG studies revealed elongated distal motor latencies, low compound motor action potential amplitudes, and decreased rates of F-waves. Albumino-cytologic dissociation was evident in CSF studies (3 WBC/μL and 85 mg/dL protein). The elevated titers of anti-GM2 antibodies and AChR antibodies (>8 nmol/L) was detected. Thus, GBS was finally diagnosed. The symptoms were slowly improved after PE. Two months later, he recovered his strength and was successfully independent from the ventilator. Later on he was moved to the rehabilitation center for further rehabilitation in combination with oral pyridostigmine, prednisone and azathioprine (Hughes 4). However, the follow-up EMG studies were not available.

## 3 Discussion

### 3.1 Case analysis

All the 3 patients developed GBS on the ground of MG. Although symptoms of each case were not identical, cardinal features were flaccid paralysis and dyspnea, except the peripheral facial paralysis in the patient 1. The changes in the EMG and CSF studies were highly diagnostic in recognizing GBS. The elevated titers of anti-ganglioside antibodies in all the cases were suggestive of autoimmunity directed to the peripheral nerves. All the 3 cases have obtained variant recoveries. Once GBS occurred, the symptoms of GBS were often severe and the recovery was generally not satisfactory. The patient 2 was much older than the patients 1 and 3, but he was completely relieved from GBS, indicating that the old age maybe not an unfavorable factor for prognosis. EMG findings on the patients 1 and 3 were much more seriously changed in the peripheral nerves than on the patient 2. Severe changes in EMG studies may herald a worse outcome. The type C of thymoma in patient 3 may constitute a factor for the worst outcome. However, the factors influencing the outcome of GBS occurred in MG patients are not fully known.

### 3.2 Literature review

In all, there were 21 reports stating 22 cases who had combined MG and GBS reviewed through PubMed, China Knowledge Network and Wangfang Medical database search from 1982 to 2018. These include 7 reports published in Chinese (Table 1),^[2–8]^ 14 reports on 15 cases published in English,^[1,9–21]^and 3 our cases (Table 2). There were another 2 reports of MG combined with chronic inflammatory demyelinating polyneuropathy,^[22,23]^ and 1 report of MG combined with possible GBS or MG presenting like GBS.^[24]^ The 25 patients (19 males and 6 females) aged from 17 to 90 years old, with a predominance in males. For the sequence of occurrence of the 2 disorders, 9 patients developed GBS after MG, and 9 occurred simultaneously. Infections were the most important predisposing factors. In the weeks to months before GBS, 13 patients had upper respiratory tract infection or pulmonary infection and 2 patients had diarrhea. Cranial nerve involvement was relatively less frequent, for only 7 cases had facial paralysis. Concerning the variants of GBS, AIDP was identified in 12 cases, AMAN in 5 cases, AMSAN in 3 cases, and MFS in 5 cases. The cardinal symptoms were pure motor disability in the majority, and only 4 cases had mild limb numbness simultaneously. Most (12/19) of the patients had normal thymus with only 3 cases of thymoma and 4 cases of thymic hyperplasia. The EMG studies showed abnormalities in 22 cases out of 24 patients, with the slowed nerve conduction velocities, decreased amplitudes of action potentials or decreased rate of F waves. Twenty out of 23 patients had albumimo-cytologic dissociation in CSF. The test of anti-ganglioside antibodies was performed in 10 cases and 9 cases had elevated titers. Most (18/20) cases had elevated titers of AChR antibodies. All (22/22) the cases had the typical changes on RNS. The employed treatments for those patients included pyridostigmine (19/22), glucocorticoids (15/22), azathioprine (3/22), cyclosporine (1/22), FK506 (1/22), IVIG (13/22), PE (4/22), and IVIG combined with PE (3/22). Functional outcomes were assessed in 22 patients according to the adopted scale by Hughes.^[25]^ The Hughes grades were: 0 = healthy, no signs or symptoms of GBS; 1 = minor symptoms or signs and able to run; 2 = able to walk >5 m without assistance, but unable to run; 3 = able to walk >5 m with assistance (1 person and waist-level walking-frame, stick, or sticks); 4 = bed- or chair-bound: unable to walk as in 3; 5 = requiring assisted ventilation or at least part of the day; 6 = dead. The prognosis was relatively favorable in 13 cases (Hughes 0–1). However, 2 patients died from the complications.

**Table 1:**
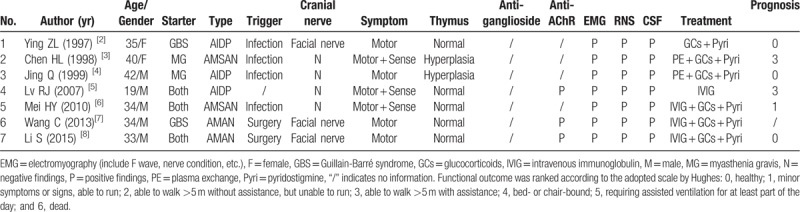
The features of comorbid MG and GBS in Chinese literature (7 cases).

**Table 2:**
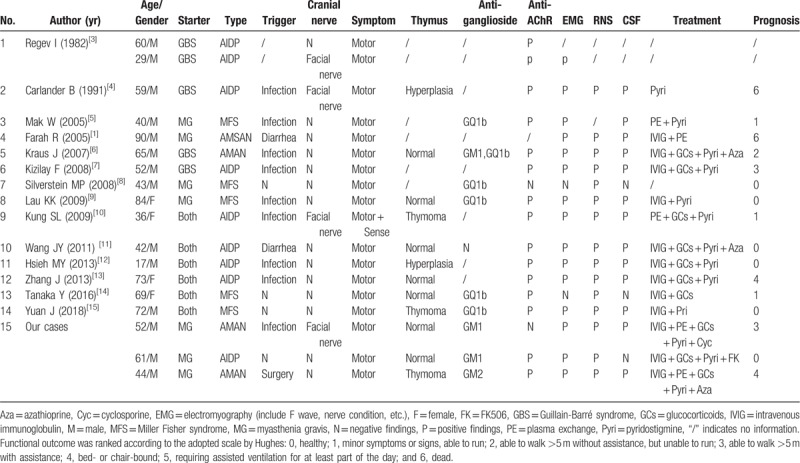
The features of comorbid MG and GBS in English literature (18 cases in 15 reports).

### 3.3 Case discussion

In our report, all the patients were males, aged 44, 52, and 61 years old, respectively. They all had newly GBS symptoms after the diagnosis of MG. The identified triggers of them were infection and surgery. One patient presented with cranial nerve involvement (facial paralysis). Interestingly, all of our 3 patients presented with pure motor symptoms without obvious sensory changes, which were consistent with the most literature.^[1,9–15,17–21]^ The presence of anti-GM antibodies may explain the pure motor deficits in these cases.^[12]^ Whether the presence of anti-GM and anti-AChR antibodies at the same time was the results of the stander-by effect needs more studies. The areflexia paralysis without fluctuation may be the key clinical clue for GBS, which is not typical for MG weakness. This is how to distinguish the pure motor form of GBS from the deteriorated generalized MG. However, typical changes in EMG studies, and albumino-cytologic dissociation in CSF were pivotal for the confirmation of GBS. The anti-GM antibodies in serum are currently considered as markers for autoimmune damages to the peripheral nerves.^[26]^.

### 3.4 Pathogenesis

A few types of GBS including AMAN, AMSAM, and MFS were reported to be combined with MG. However, the detailed pathogenesis of the 2 combined diseases remained elusive. The first experimental model of MG combined with polyneuropathy was reported by Festoff et al in 1977.^[27]^ They suggested that the release of axonal protoplasmic proteins activated the autoimmune response and resulted in polyneuropathy and myasthenia-like syndrome. Until now, there have been only few cases reports and 3 literature review studies concerning MG combined with GBS.^[18,19,21]^ The hypotheses include:

(1) molecular mimicry. A crossed antigen produced by pathological factors, such as infections, which acted on the AChR at the end-plates on muscles and sphingomyelin of peripheral nerve. This may cause autoimmune attacks, which was in turn, resulting in presentations of both MG and GBS at the same time.^[28]^ This idea has been confirmed by an animal model established by Krampfl et al^[29]^;
(2) there is a possibility that there are some degrees of similarity between AChR and peripheral nerves. A type of IgG antibodies could simultaneously damages both AChR and peripheral nerves, bringing about MG and GBS in the same patient. Thus the IgG antibodies in patients with MG combined GBS may significantly impede the transmission of the AChR channel, resulting in muscle weakness.^[29]^ This hypothesis could explain why the 2 diseases recover rapidly after PE. Silverstein's study^[8]^ demonstrated the electrophysiological abnormalities in the neuromuscular junction of MG and MFS;
(3) the long-term MG course may play a role in slowly activating the immune system. Any infections or other incentives could make the body more likely to produce antibodies to peripheral nerves causing GBS.^[16]^ Meanwhile, a previously activated immune system may generate autoantibodies to attack other antigens^[15]^;
(4) thymic abnormalities such as thymoma or hyperplasia in MG patients may play important roles in the development of other autoimmune diseases.^[30–32]^ Any triggers such as infections would accelerate the activated immune system to produce more specific antibodies. That may reach the threshold to uncover presentations of both GBS and MG.^[16]^

## 4 Conclusion

MG combined with GBS is rare, and the pathogenesis is not fully appreciated yet. Muscle weakness involving extremities, extra-ocular and bulbar muscles are the cardinal manifestations of both MG and GBS. Although their characteristics are quite different, there are still some difficulties in making a correct diagnosis when the 2 diseases happened in 1 same patient. Careful and comprehensive examinations will minimize ignorance of recognizing GBS in MG patients. Early recognition of the 2 diseases and proper management are vital for a better prognosis.

## Acknowledgments

We greatly appreciate the patients and their family for signing the informed consent for publishing the data.

## Author contributions

**Conceptualization:** Bitao Bu.

**Investigation:** Mengcui Gui.

**Project administration:** Suqiong Ji.

**Writing – review & editing:** Bitao Bu.

**Writing – original draft:** Yayun Cao.
